# Gene expression meta-analysis identifies metastatic pathways and transcription factors in breast cancer

**DOI:** 10.1186/1471-2407-8-394

**Published:** 2008-12-30

**Authors:** Mads Thomassen, Qihua Tan, Torben A Kruse

**Affiliations:** 1 Department of Biochemistry, Pharmacology, and Genetics, Odense University Hospital and Human Microarray Centre (HUMAC), University of Southern Denmark, Odense, Denmark; 2 Institute of Public Health, University of Southern Denmark, Odense, Denmark

## Abstract

**Background:**

Metastasis is believed to progress in several steps including different pathways but the determination and understanding of these mechanisms is still fragmentary. Microarray analysis of gene expression patterns in breast tumors has been used to predict outcome in recent studies. Besides classification of outcome, these global expression patterns may reflect biological mechanisms involved in metastasis of breast cancer. Our purpose has been to investigate pathways and transcription factors involved in metastasis by use of gene expression data sets.

**Methods:**

We have analyzed 8 publicly available gene expression data sets. A global approach, "gene set enrichment analysis" as well as an approach focusing on a subset of significantly differently regulated genes, GenMAPP, has been applied to rank pathway gene sets according to differential regulation in metastasizing tumors compared to non-metastasizing tumors. Meta-analysis has been used to determine overrepresentation of pathways and transcription factors targets, concordant deregulated in metastasizing breast tumors, in several data sets.

**Results:**

The major findings are up-regulation of cell cycle pathways and a metabolic shift towards glucose metabolism reflected in several pathways in metastasizing tumors. Growth factor pathways seem to play dual roles; EGF and PDGF pathways are decreased, while VEGF and sex-hormone pathways are increased in tumors that metastasize. Furthermore, migration, proteasome, immune system, angiogenesis, DNA repair and several signal transduction pathways are associated to metastasis. Finally several transcription factors e.g. E2F, NFY, and YY1 are identified as being involved in metastasis.

**Conclusion:**

By pathway meta-analysis many biological mechanisms beyond major characteristics such as proliferation are identified. Transcription factor analysis identifies a number of key factors that support central pathways. Several previously proposed treatment targets are identified and several new pathways that may constitute new targets are identified.

## Background

Breast cancer is the most common cancer among women and the leading cause of cancer related death. Metastasis is the main cause of death of the disease but the knowledge of biological mechanisms in metastasis is still fragmentary. Risk of recurrence is evaluated by clinical and pathological criteria. However, the performance of this method is far from optimal. Gene expression profiling of tumors has been used for classification of cancer outcome in several studies with promising results for improvement of risk prediction [1-9]. Despite these promising clinical results, little insight to biological mechanisms has been obtained from the large amount of gene expression data. Pathway or gene ontology analyses in these studies are limited to genes in the outcome classifier. The main mechanism observed is up regulation of cell cycle while other pathways are more inconsistent between the studies[5,10-12]. Another potential use of these data is to map deregulation of transcription factors and miRNA having impact on metastasis. If sufficient knowledge about sequence motifs for these regulators existed, groups of genes with common motifs might display concordant deregulation in metastasizing tumors. An extensive knowledge of transcription factor binding elements is gathered in TransFac database  and bioinformatic prediction of target genes has been useful, while prediction of miRNA target genes is still in its infancy [13]. However, systematic investigation with these methods in prognostic gene expression data sets has not been performed for breast cancer. The combined knowledge of pathways and regulators of gene transcripts may deepen the understanding of biological mechanisms in metastasis.

Prognostic data set have been used for validation of prognostic significance of gene sets e.g. representing a certain pathway. For example Bild et al. developed cell models representing candidate metastatic pathways, defined characteristic gene sets by microarray gene expression analysis, and demonstrated prognostic value in the prognostic data sets performed with gene expression [14]. However, these candidate pathways presumably only represent a fraction and not necessarily the most important of the pathways involved in metastasis. We hypothesize that many biological pathways and transcriptional regulators are involved in metastasis of breast cancer and that they are reflected in gene expression patterns of primary tumors. Our aim is to elucidate metastatic pathways and secondly transcriptional regulators and to integrate the knowledge of these informations.

Pathway analysis programs typically compare level of gene expression in two sets of samples by ranking genes according to a statistic model and apply a cut-off value for genes resulting in a group of significantly up- and down-regulated genes. Overrepresentation analysis then identifies pathways significantly regulated in these groups. We have supplemented this kind of analysis with a more global approach by gene set enrichment analysis (GSEA), to identify pathways that are differentially regulated in metastasizing and non-metastasizing tumors. By this method, the imbalance of pathway gene-sets is examined in the entire list of ranked genes. In a single data set this analysis will generally not result in significant findings beyond major pathways like cell cycle. By performing meta-analysis of 8 data sets, we have increased the power to identify pathways and transcription factors involved in metastasis of breast cancer.

## Methods

### Data sets

Eight publicly available data sets of gene expression at RNA level in primary tumors were included in the analysis. These studies are performed with different platforms, different populations etc. as depicted in table 1. The outcome differs in that local and regional recurrences are included in some studies. However, non-metastatic relapse constitute a minority in clinical cohorts. There may be overlap in the samples in the different data set e.g. samples from Uppsala in Sotiriou 2006 and Uppsala data sets, but the total number of different tumor samples is at least 1200.

**Table 1:** Characteristics of patients and platforms in included studies.

**Data set**	**chip**	**# probes**	**Patients, country, nodal status^a^**	**outcome^b^**	**Adjuvant systemic treatment^c^**
HUMAC [1]	spotted oligonucleotides	29K	n = 60, DK N-, low-malignant	metastasis	nil

Huang [2]	Affymetrix 95av2	12K	n = 52, Taiwan N+	relapse	ct

Sotiriou 2003 [3]	Spotted cDNA	7.6K	n = 99, UK N+/N-	relapse	et, ct

Sotiriou 2006 [4]	Affymetrix HG-133A	22K	n = 179 S (Uppsala), UK N+/N-	dm	et

Rotterdam [5]	Affymetrix HG-133A	22K	n = 286, NL N-	dm	nil

Amsterdam [6]	Rosetta	25K	n = 295, NL N+/N-	dm	nil, ct, et

Uppsala [7]	Affymetrix HG133A+B	44K	N = 236, S (Uppsala) N+/N-	death from breast cancer	nil, ct, et

Stockholm [8]	Affymetrix HG-133A+B	44K	n = 159, S (Stockholm) N+/N-	relapse	nil, ct, et

a: n, number of patients included; N+, positive nodal status; N-, negative nodal status; DK, Denmark; UK, United Kingdom; NL, the Netherlands; S, Sweden.

b: dm, distant metastasis,

c: ct, chemotherapy, et: endocrine therapy

The normalizations performed in the studies were retained because the authors found these methods optimal for the data sets, and because pathway analysis was performed separately in each data set.

### Gene set enrichment analysis of pathways, transcription factors and miRNA

GSEA v 2.0 [15] was used with 450 curated gene sets representing individual pathways. These pathway gene sets are adopted from KEGG , GenMapp , Biocarta  etc. and gathered in the Molecular Signature Database implemented in GSEA. For the analysis of promoter and miRNA response elements 837 sequence elements predicted or validated to bind transcription factors or miRNA's were downloaded from MsigDB. The motifs are collected from TransFac  and mirBase  and target gene sets defined by bioinformatic prediction of target genes [13,15]. The GSEA program ranks genes according to a signal-to-noise value: (X_A_-X_B_)/(s_A_+s_B_), where X is the mean and s is the standard deviation for the two classes A and B (metastases and non-metastases). When several probes recognized the same gene, the probe with max expression value was extracted using the "collapse to gene set" function. Gene sets represented by less than 15 genes in a data set was excluded except for the Sotiriou 2003 data set where this threshold was set to 10 genes because of the low number of genes on that chip.

The output from GSEA is an enrichment score, describing the imbalance in the distribution of ranks of gene expression in each gene set between metastasizing and non-metastasizing tumors. The enrichment score is normalized according to size of the gene sets. Then, gene set were ranked according to the normalized enrichment score with gene sets up-regulated in metastasizing tumors on the top and down-regulated gene sets in the bottom.

### GenMAPP pathway analysis

To perform pathway analysis with an independent method, GenMapp 2.0 software was used, by applying a significance cut-off for genes between metastasizing and non-metastasizing tumors of p = 0.05 in Students T-test. The software uses Fisher exact test to examine overrepresentation of up-regulated or down-regulated genes among 203 pathways. The output is a ranked list of up-regulated and down-regulated pathways respectively for each data set.

### Meta-analysis

The ranked lists of gene sets for each analysis generated by GSEA or GenMAPP from the 8 data sets were integrated so that only gene sets represented in output from all data sets were included. The initial 450 pathway gene sets in MSigDb for the GSEA pathway analysis were reduced to 223 gene sets passing the threshold (at least 10 or 15 genes in gene sets) in all data sets. For analysis of transcription factor and miRNA binding sites, 837 motif gene sets from MSigDb were reduced to 761 motif gene sets. 203 GenMAPP pathways were reduced to 177 pathways present in all data sets. For each data set, individual gene sets were assigned a ranking value from 1 to the maximum number of gene sets, according to the ranking performed by GSEA or GenMAPP. The mean ranking value for each gene set was calculated across the data sets and finally the gene sets were ranked according to this value.

Our null-hypothesis is that the expressions of genes in pathway gene sets are unrelated to metastasis. This means that the ranking value for a given gene set in a given data set is expected to be a random value between 1 and the maximum number of gene sets analyzed. To simulate the distribution of mean ranking values across the 8 data sets fulfilling the null-hypothesis, random drawing of 8 ranking values were performed 10^6 ^times and the mean value was calculated each time. A null distribution of mean ranking values was generated from these results. To test the significance for a given gene set, the observed mean ranking value was compared to the null distribution. To fulfill the null-hypothesis an observed mean ranking value should be within 95% interval of the null-distribution. This calculation, estimation of p-value and correction of p-value by false discovery rate (FDR) was performed in R environment . Gene sets with FDR values below 0.05 were considered significant.

## Results

### Pathway analysis

Data from more than 1200 breast cancer patients were collected (table 1). GSEA only identified few significant pathways within each data set (data not shown). However, by performing meta-analysis of gene sets ranked by enrichment score, several gene sets turned out to have low ranking number in the majority of data sets indicating up-regulation of corresponding pathway in metastasizing tumors compared to non-metastasizing tumors. Similar, gene sets with a high mean ranking value indicated low expression in metastasizing tumors compared to non-metastasizing tumors (table 2). False discovery rates indicated 38 of these gene sets to be significantly differentially expressed; 26 up-regulated and 12 down-regulated. The most striking pathways are DNA replication and cell cycle that are both up-regulated.

**Table 2:** Metastatic pathways identified by gene set enrichment meta-analysis.

**Pathway**	**Ams**	**Hua**	**HUM**	**Rot**	**S03**	**S06**	**Sto**	**Upp**	**mean**	**p**	**FDR**
**Up-regulated**											
DNA_REPLICATION_REACTOME	11	64	2	19	5	6	2	6	14.4	< 10E-6	< 10E-6
CELL_CYCLE_KEGG	4	101	1	14	39	5	3	12	22.4	1.0E-6	1.1E-4
ATRBRCAPATHWAY	58	10	23	1	73	41	9	17	29.0	2.2E-5	1.5E-3
AMINOACYL_TRNA_BIOSYNTHESIS	35	90	57	21	9	9	8	10	29.9	2.7E-5	1.5E-3
PYRIMIDINE_METABOLISM	5	9	11	20	159	3	7	34	31.0	3.5E-5	1.6E-3
G1_TO_S_CELL_CYCLE_REACTOME	22	141	5	5	65	19	5	11	34.1	8.2E-5	3.0E-3
PROTEASOME_DEGRADATION	29	128	71	29	2	13	1	7	35.0	1.0E-4	3.0E-3
G2PATHWAY	30	107	4	23	86	8	15	9	35.3	1.1E-4	3.0E-3
PROTEASOMEPATHWAY	36	155	45	10	1	14	10	16	35.9	1.3E-4	3.1E-3
PURINE_METABOLISM	23	70	54	38	57	22	22	28	39.3	2.7E-4	6.0E-3
MRNA_PROCESSING_REACTOME	59	67	68	34	14	53	23	20	42.3	5.0E-4	1.0E-2
PROTEASOME	38	171	18	31	3	50	12	26	43.6	6.6E-4	1.2E-2
VEGFPATHWAY	41	52	84	4	37	38	81	13	43.8	6.8E-4	1.2E-2
PENTOSE_PHOSPHATE_PATHWAY	28	140	83	68	10	17	4	3	44.1	7.3E-4	1.2E-2
CELLCYCLEPATHWAY	44	112	3	13	139	2	27	21	45.1	8.7E-4	1.3E-2
GLYCOLYSIS_AND_GLUCONEOGENESIS	1	145	107	62	4	10	16	24	46.1	1.1E-3	1.5E-2
OXIDATIVE_PHOSPHORYLATION	80	142	25	15	85	16	11	1	46.9	1.2E-3	1.6E-2
G1PATHWAY	75	39	27	11	171	25	36	8	49.0	1.7E-3	2.0E-2
ARAPPATHWAY	19	8	175	17	49	43	25	56	49.0	1.7E-3	2.0E-2
FRUCTOSE_AND_MANNOSE_METABOLISM	10	11	145	41	58	101	19	14	49.9	2.0E-3	2.3E-2
S1P_SIGNALING	17	35	60	25	131	66	51	22	50.9	2.4E-3	2.5E-2
ACTINYPATHWAY	6	219	50	2	15	60	24	43	52.4	3.0E-3	3.1E-2
ELECTRON_TRANSPORT_CHAIN	131	191	36	9	41	12	6	5	53.9	3.8E-3	3.7E-2
RNA_TRANSCRIPTION_REACTOME	133	69	6	24	97	49	37	19	54.3	4.0E-3	3.7E-2
MPRPATHWAY	26	176	12	28	129	1	20	44	54.5	4.2E-3	3.7E-2
UBIQUITIN_MEDIATED_PROTEOLYSIS	46	125	128	89	6	15	14	15	54.8	4.3E-3	3.7E-2
											
**Down-regulated**											
HISTIDINE_METABOLISM	193	151	117	189	184	191	145	212	172.8	2.5E-3	4.6E-2
PPARAPATHWAY	192	167	131	177	168	133	206	210	173.0	2.4E-3	4.6E-2
GLYCEROLIPID_METABOLISM	165	85	204	207	221	185	155	177	174.9	1.8E-3	4.0E-2
FATTY_ACID_METABOLISM	164	210	176	138	200	140	163	211	175.3	1.7E-3	4.0E-2
PDGFPATHWAY	209	127	182	185	112	181	212	213	177.6	1.1E-3	3.1E-2
EGFPATHWAY	208	124	192	191	106	197	190	215	177.9	1.1E-3	3.1E-2
NUCLEAR_RECEPTORS	173	202	121	174	199	150	214	193	178.3	9.8E-4	3.1E-2
BETA_ALANINE_METABOLISM	218	164	116	165	218	163	193	203	180.0	7.2E-4	3.1E-2
TOLLPATHWAY	212	119	167	204	128	223	165	222	180.0	7.2E-4	3.1E-2
GPCRDB_OTHER	134	196	164	158	210	196	205	195	182.3	4.5E-4	3.1E-2
GLEEVECPATHWAY	219	73	177	219	203	166	199	219	184.4	2.9E-4	3.1E-2
VALINE_LEUCINE_AND_ISOLEUCINE_DEGRADATION	222	174	184	181	207	194	97	217	184.5	2.8E-4	3.1E-2

The ranking numbers indicate the ranking of each gene set (pathway) out of the 223 gene sets in each data set and the mean ranking number indicate the ranking in the meta-analysis. Only 38 significant out of a total of 223 gene sets are shown. Ams: Amsterdam, Hua: Huang, HUM: HUMAC, Rot: Rotterdam, S03: Sotiriou 2003, S06: Sotiriou 2006, Sto: Stockholm, Upp: Uppsala.

An independent method, GenMAPP, with a very different approach, including only the most significant genes, was applied to the same data (table 3). In this approach, analysis of up- and down-regulated gene sets are performed independently, resulting in two ranked output lists, with up- or down-regulated pathways on the top respectively. For this reason up-regulated as well as down-regulated pathways have low mean ranking numbers (table 3). Eleven pathways are up-regulated and 8 are down-regulated. Again cell cycle (KEGG and G1_to_S_control_reactome) and DNA replication reactome, but also electron transport chain, are strikingly up-regulated.

**Table 3:** Pathways identified by GennMAPP analysis

**Pathway**	**Ams**	**Hua**	**HUM**	**Rot**	**S03**	**S06**	**Sto**	**Upp**	**mean**	**p**	**FDR**
**Up-regulated**											
Hs_Cell_cycle_KEGG	1	97	2	1	1	4	1	1	13.5	< 10E-6	9.0E-5
Hs_Cell_Cycle-G1_to_S_control_Reactome	3	51	21	13	5	7	8	11	14.9	< 10E-6	9.0E-5
Hs_DNA_replication_Reactome	2	124	3	8	6	10	2	3	19.8	5.0E-6	3.0E-4
Hs_Electron_Transport_Chain	73	9	34	2	35	11	4	2	21.3	1.0E-5	4.4E-4
Hs_Androgen-Receptor_NetPath_2	82	12	9	16	20	5	43	19	25.8	4.6E-5	1.3E-3
Hs_1-Tissue-Embryonic_Stem_Cell	6	157	1	4	22	3	13	4	26.3	5.2E-5	1.3E-3
Hs_mRNA_processing_Reactome	45	22	64	6	33	26	9	5	26.3	5.2E-5	1.3E-3
Hs_Citrate_cycle_TCA_cycle_	18	23	81	40	7	35	11	21	29.5	1.5E-4	3.3E-3
Hs_Aminoacyl_tRNA_biosynthesis	19	101	84	32	10	13	3	8	33.8	4.7E-4	9.3E-3
Hs_Cholesterol_Biosynthesis	28	119	7	41	13	56	6	24	36.8	1.0E-3	1.8E-2
Hs_Hedgehog_Netpath_10	41	5	4	21	99	18	76	65	41.1	2.7E-3	4.3E-2
											
**Down-regulated**											
Hs_Adipogenesis	4	22	41	43	16	58	2	1	23.4	2.5E-5	4.4E-3
Hs_EGFR1_NetPath_4	5	3	42	44	24	13	13	80	28.0	1.0E-4	8.9E-3
Hs_T-Cell-Receptor_NetPath_11	37	2	75	6	38	28	19	51	32.0	3.0E-4	1.8E-2
Hs_Smooth_muscle_contraction	30	51	36	23	34	50	12	44	35.0	6.6E-4	2.4E-2
Hs_Insulin_Signaling	15	11	54	64	39	47	16	39	35.6	7.7E-4	2.4E-2
Hs_IL-6_NetPath_18	16	8	163	3	41	7	26	23	35.9	8.1E-4	2.4E-2
Hs_IL-7_NetPath_19	7	5	141	9	19	15	28	78	37.75	1.3E-3	3.0E-2
Hs_IL-3_NetPath_15	58	7	56	18	32	29	44	61	38.125	1.4E-3	3.0E-2

The ranking numbers indicate the ranking of each gene set (pathway) out of the 177 gene sets in each data set and the mean ranking number indicate the ranking in the meta-analysis. Only 19 significant out of a total of 177 gene sets are shown. Ams: Amsterdam, Hua: Huang, HUM: HUMAC, Rot: Rotterdam, S03: Sotiriou 2003, S06: Sotiriou 2006, Sto: Stockholm, Upp: Uppsala.

### Regulatory motifs

GSEA was also applied to gene sets having in common recognition elements for transcription factors within promoter regions or miRNA recognition elements in 3'UTR's (table 4). Gene sets with low mean ranking number and FDR below 0.05 were interpreted as significantly up-regulated and gene sets with high mean ranking number were considered down-regulated. In this analysis it is striking that all significant gene sets are up-regulated and E2F family members are strongly overrepresented. Several different gene sets predicted to be recognized by the same transcription factor are included in the analysis because different variants of binding motifs have been reported to TransFac database. Elements with unknown transcription factor are predicted by bioinformatic comparison of promoter regions [13].

**Table 4:** Gene set enrichment meta-analysis of transcriptional regulatory motifs

**motif**	**Ams**	**Hua**	**HUM**	**Rot**	**S03**	**S06**	**Sto**	**Upp**	**mean**	**p**	**FDR**
V$E2F_Q6_01	2	165	13	75	2	11	16	25	38.6	< 10E-6	< 10E-6
V$E2F_03	3	273	15	25	7	13	13	9	44.8	< 10E-6	< 10E-6
V$E2F_Q4_01	7	235	17	65	4	20	6	16	46.3	< 10E-6	< 10E-6
KTGGYRSGAA_UNKNOWN	16	191	8	126	43	1	8	23	52.0	< 10E-6	< 10E-6
V$E2F_Q3	30	289	22	80	23	29	4	8	60.6	< 10E-6	< 10E-6
V$E2F1_Q4_01	18	501	18	12	51	24	14	4	80.3	3.0E-6	3.3E-4
V$E2F1_Q6_01	15	440	14	79	44	38	11	14	81.9	3.0E-6	3.3E-4
V$E2F_Q3_01	14	535	21	14	46	28	15	5	84.8	6.0E-6	5.3E-4
V$E2F_Q6	28	595	9	17	9	15	2	6	85.1	7.0E-6	5.3E-4
GCCATNTTG_V$YY1_Q6	89	87	254	60	27	117	39	29	87.8	7.0E-6	5.3E-4
V$E2F1_Q3	9	588	5	43	35	21	7	2	88.8	8.0E-6	5.5E-4
V$E2F_Q4	26	617	12	16	10	25	3	7	89.5	1.0E-5	6.3E-4
V$E2F1DP1RB_01	21	607	10	34	19	16	9	3	89.9	1.2E-5	7.0E-4
ACTWSNACTNY_UNKNOWN	218	32	142	151	90	63	10	48	94.3	1.5E-5	8.2E-4
GGAANCGGAANY_UNKNOWN	227	14	305	58	45	110	1	1	95.1	1.7E-5	8.6E-4
TCCCRNNRTGC_UNKNOWN	151	117	33	10	152	158	33	115	96.1	1.8E-5	8.6E-4
V$NFY_Q6	84	74	36	272	151	76	25	66	98.0	2.2E-5	9.8E-4
V$E2F1_Q6	32	649	6	36	21	12	12	19	98.4	2.5E-5	1.1E-3
V$E2F4DP1_01	31	672	7	23	13	19	18	15	99.8	3.0E-5	1.1E-3
SGCGSSAAA_V$E2F1DP2_01	4	698	11	26	12	17	17	18	100.4	3.0E-5	1.1E-3
V$E2F1DP2_01	11	695	1	44	15	6	21	11	100.5	3.1E-5	1.1E-3
V$E2F1DP1_01	12	694	2	45	16	7	22	12	101.3	3.2E-5	1.1E-3
V$E2F4DP2_01	13	693	3	46	17	8	23	13	102.0	3.5E-5	1.1E-3
V$E2F_02	24	685	4	48	18	10	20	10	102.4	3.5E-5	1.1E-3
TGASTMAGC_V$NFE2_01	19	166	301	152	6	23	83	87	104.6	4.1E-5	1.2E-3
AACYNNNNTTCCS_UNKNOWN	77	9	299	216	22	101	52	77	106.6	4.6E-5	1.3E-3
V$NRF1_Q6	38	224	114	233	8	150	51	42	107.5	4.7E-5	1.3E-3
V$ELK1_02	198	219	160	32	194	56	27	26	114.0	7.7E-5	2.1E-3
V$E2F1_Q4	25	511	39	98	96	85	26	45	115.6	9.1E-5	2.4E-3
GCGSCMNTTT_UNKNOWN	208	56	37	396	37	161	48	49	124.0	1.8E-4	4.4E-3
V$GABP_B	36	22	173	562	86	107	5	17	126.0	2.1E-4	5.0E-3
V$YY1_Q6	216	215	304	87	26	81	58	59	130.8	2.8E-4	6.5E-3
V$USF2_Q6	172	207	82	328	113	53	57	53	133.1	3.2E-4	7.4E-3
V$NRF2_01	301	19	383	64	103	127	74	39	138.8	4.6E-4	1.0E-2
V$E2F_01	20	641	16	222	172	3	19	21	139.3	4.7E-4	1.0E-2
V$SP1_Q6_01	23	18	310	545	29	102	35	61	140.4	4.9E-4	1.0E-2
V$ARNT_02	221	248	68	169	76	222	38	86	141.0	5.1E-4	1.0E-2
V$HIF1_Q5	62	279	168	159	285	69	71	38	141.4	5.2E-4	1.0E-2
RACTNNRTTTNC_UNKNOWN	315	6	279	119	211	82	94	27	141.6	5.3E-4	1.0E-2
V$BACH1_01	17	118	349	187	133	44	63	239	143.8	6.2E-4	1.2E-2
ATCMNTCCGY_UNKNOWN	40	428	56	51	321	14	133	113	144.5	6.4E-4	1.2E-2
V$AP1_01	51	85	177	287	42	43	120	361	145.8	6.9E-4	1.2E-2
ACTAYRNNNCCCR_UNKNOWN	171	13	83	66	637	109	29	72	147.5	7.5E-4	1.3E-2
V$ER_Q6_02	118	366	63	176	177	214	37	58	151.1	9.4E-4	1.6E-2
V$CETS1P54_01	308	55	244	134	317	41	80	36	151.9	9.8E-4	1.7E-2
V$NFY_01	1	559	42	157	106	242	30	92	153.6	1.1E-3	1.8E-2
V$COUP_DR1_Q6	86	67	194	232	269	223	62	97	153.8	1.1E-3	1.8E-2
V$NFY_C	73	135	35	539	245	86	47	90	156.3	1.2E-3	2.0E-2
V$AP4_Q6_01	60	228	79	459	250	57	75	91	162.4	1.7E-3	2.6E-2
CTCNANGTGNY_UNKNOWN	199	43	30	259	255	116	251	167	165.0	1.9E-3	3.0E-2
TMTCGCGANR_UNKNOWN	550	485	72	59	68	60	24	24	167.8	2.2E-3	3.3E-2
V$MYCMAX_B	22	510	271	276	234	4	36	34	173.4	2.9E-3	4.2E-2
YGCGYRCGC_UNKNOWN	67	267	249	491	160	88	41	28	173.9	2.9E-3	4.2E-2
V$AP2_Q3	78	58	91	544	279	96	111	137	174.3	3.0E-3	4.2E-2
CCAWNWWNNNGGC_UNKNOWN	39	256	222	203	360	153	100	93	178.3	3.5E-3	4.9E-2

Genes with common transcriptional regulatory sequence elements constitute a gene set. The ranking numbers indicate the ranking of each gene set out of a total of 761 gene sets in each data set and the mean ranking number indicate the ranking in the meta-analysis. Only 55 significantly up-regulated out of a total of 761 gene sets are shown. No gene sets were significantly down-regulated. Ams: Amsterdam, Hua: Huang, HUM: HUMAC, Rot: Rotterdam, S03: Sotiriou 2003, S06: Sotiriou 2006, Sto: Stockholm, Upp: Uppsala.

## Discussion

We have performed meta-analysis of 8 publicly available gene expression data sets to identify common biological mechanisms involved in metastasis of breast cancer. The identified pathways can be grouped into a limited number of categories: cell cycle and proliferation, growth factor pathways, metabolism, angiogenesis, gleevec, migration, signal transduction, proteasome pathway, immune system, and DNA damage sensing and repair, which are discussed in section 1 below. Some of the pathways are supported by key transcription factors identified by motif analysis. In section 2, the methods used for pathway analysis and the advantage of meta-analysis are discussed.

### 1.1 Cell cycle and proliferation

Among the pathways most up-regulated in metastasizing tumors, identified by GSEA, are cell cycle pathways represented by five cognate pathways (CELL_CYCLE_KEGG, CELLCYCLEPATHWAY, G1PATHWAY, G1_TO_S_CELL_REACTOME, and G2PATHWAY). This is supported by GenMAPP analysis identifying two of these pathways to be most differently regulated between metastasizing and non-metastasizing tumors (CELL_CYCLE_KEGG and G1_TO_S_CELL_REACTOME). Up-regulation of cell cycle and proliferation is a hallmark of cancer cells compared to normal cells. Our observation, that cell cycle genes are up-regulated in cells with metastatic potential compared to non-metastatic cells, is in agreement with some previously studies demonstrating cell cycle as the major up-regulated pathway in metastasizing breast cancer [5,10]. Up-regulation of DNA_REPLICATION_REACTOME is required for cell division and is sustained by both GSEA and GenMAPP. Up-regulation of Purine and pyrimidine metabolism pathways most likely also reflect elevated biosynthesis of DNA, and pentose phosphate pathway serves to generate ribose 5-phosphate that is a precursor in nucleotide synthesis and NADPH that donates electrons for biosynthesis of several molecules. Furthermore, pathways involved in transcription (RNA_TRANSCRIPTION_REACTOME, mRNA_PROCESSING_REACTOME) and translation (AMINOACYL_TRNA_BIOSYNTHESIS) are up-regulated. The crucial role of cell cycle and proliferation genes in metastasis is strongly supported by several up-regulated gene sets with recognition sites for E2F identified by motif analysis (table 4). This family of transcription factors have several targets involved in cell cycle e.g. cyclin E [16] and expression of some E2F family members have been associated with poor prognosis in breast carcinomas [17]. Furthermore, NRF1 a transcription factor that co regulates a large number of E2F target genes [18] and YY1 associated with unchecked cellular proliferation [19] are also up-regulated according to the motif analysis.

### 1.2 Growth factors

Two growth factor pathways are down-regulated in tumors with metastatic potential: Epidermal growth factor (EGF) and platelet-derived growth factor (PDGF) pathways. This is surprising and one might expect the opposite findings in cells with elevated proliferation. EGFR is known to transmit mitogenic signals from EGF and TGFα to several downstream signaling cascades: Phospholipase C, RAS, phosphatidylinositol-3 kinase (PI-3K), and STAT's. However, emerging evidence suggests an alternative mechanism that involves transport of activated EGFR from the cell membrane to the nucleus and direct association with gene promoters. Among the targets for nuclear EGFR is *cyclin D1 *and *iNOS *[20]. The present results indicate down-regulation of conventional mechanism which might reflect activation of nuclear localization pathway. The majority of genes in the EGF gene set are functioning in the second messenger system while the nuclear localization pathway only involves EGFR. *EGFR *itself is not differentially regulated (data not shown), but it might be activated by other mechanisms. However, the activity of the transcription factor ELK1 that is downstream target in the conventional pathway is up-regulated according to motif analysis indicating complex alterations of the EGF pathway. Conflicting results have been reported for the prognostic significance of EGF expression in breast cancer (reviewed by Chan et al [21]).

PDGF signaling has previously been linked with breast cancer metastasis in a mouse model. Furthermore, increased expression of PDGFR and other key molecules has been measured in invasive carcinomas compared to intra ductal carcinomas and normal tissue [22]. This is conflicting with present results showing overall down-regulation of PDGF pathway. The oncogene *RAS *which gene product is activated by among others PDGF is however, up-regulated in 5 of 7 data sets (data not shown). The explanation for this discrepancy is unclear. The results indicate differential expression of several genes between metastasizing and non-metastasizing human breast tumors, while other mechanisms may be present in mice models and in the non-metastatic progression of tumors. Other mechanism than the traditional signal transduction pathways might also be involved. Furthermore, methodological problems for example in the definition of gene sets may have impact. Other mechanisms than gene regulation are surely involved in these pathways.

### 1.3 Metabolism

Several pathways involving basic energy metabolizing is activated in metastasizing tumor cells: Glycolysis and glyconeogenesis, citric acid cycle, oxidative phosphorylation, and electron transport chain. The pathway glycolysis and glyconeogenesis cover presumably only glycolysis, because glyconeogenesis is restricted to liver and certain other organs. Glycolysis pathway converts glucose to pyrovate which is subsequently decarboxylated in several reactions in citric acid cycle, a pathway that is up-regulated according to GenMAPP. NADH and FADH2, generated in citric acid cycle, contain electrons that are transferred to molecular oxygen in electron transport chain and oxidative phosphorylation. This results in generation of a proton gradient across mitochondrial inner membrane, driving synthesis of ATP. Fructose and mannose metabolism pathway, up-regulated according to GSEA, presumably cover the entry of fructose into glycolysis by hexokinase, a general mechanism in adipose tissue, where fructose level is high [23]. Hexokinase is believed to be central for maintaining a high glycolytic phenotype that characterizes cancer cells compared to normal cells especially under conditions of hypoxia often present in cancer cells [24]. This is supported by motif analysis indicating activation of NFY and SP1 transcription factors which has been shown to activate hexokinase [25]. Furthermore, pathways generating energy from fatty acid (fatty acid and clycerolipid metabolism) and amino acids (valine, leucine, isoleucine, beta-alanine, and histidine degradation) are down-regulated (GSEA).

These findings indicate that tumor cells with a metastatic potential derive energy, to a higher extend, from carbohydrates and to a lesser extend fatty acids and amino acids compared to non-metastasizing tumor cells. Furthermore, elevated synthesis and consumption of ATP appears to be a feature of metastasizing tumor cells. Higher glucose metabolism is also a characteristic of cancer cells, compared to normal cells, used for detection of tumors and metastases by PET scanning [26]. Glucose metabolism has been hypothesized to improve survival of cancer cells under hypoxia which is often observed in tumors [27]. Here we demonstrate prognostic disadvantage of higher glucose metabolism in primary breast tumors. Furthermore, targets for the transcription factor hypoxia inducible factor 1 (HIF1) is induced according to motif analysis, strongly supporting the recent theory that HIF1 is an inducer of glycolysis in response to hypoxia [27]. To our knowledge no previous report has linked both elevated glycolysis and decreased amino acid and fatty acid degradation to breast cancer metastasis. Furthermore, the metabolic shift is reflected in many sustaining pathways supporting the results.

### 1.4 Angiogenesis

Increased angiogenesis is believed to be an important qualification for survival of breast cancer cells during hypoxia often featuring breast tumors. Vasculature develops in response to growth factors like VEGF released by the tumor. Furthermore, VEGF receptors on tumor cells initiate a autocrine signaling response that facilitates survival in hypoxia and in response to other apoptotic stimuli [28]. This is supported by up-regulation of VEGF pathway in present data sets. VEGF-inhibitors are being tested as breast cancer drugs [29]. Furthermore, PPAR pathway is down-regulated in metastasizing tumor cells. PPARgamma is a ligand-activated transcription factor that has been associated with decreased angiogenesis and invasiveness and with increased patient survival [30-32] in agreement with present results.

### 1.5 Gleevec pathway

In chronic myelogenous leukemia (CML) the tyrosine kinase ABL is often activated by a chromosomal translocation, t(9;22), resulting in fusion of *BCR *and *ABL *genes. The kinase activity of ABL in the BCR-ABL fusion is activated and up-regulated; driving the uncontrolled cell growth observed in CML. Gleevec is developed to inhibit ABL and also inhibits the PDGF receptor tyrosine kinase and the c-kit tyrosine kinase. The gene set included in the present analysis only includes BCR-ABL pathway. Gleevec has been introduced as anti-cancer drug not only against cancer cells with BCR-ABL translocation, but also in some cancers not having the translocation [33]. However, the BCR-ABL and PDGF pathways inhibited by gleevec is down-regulated in metastasizing tumors according to our analysis, which might indicate that inhibition of ABL is not relevant for treatment of breast cancer.

### 1.6 Migration

Mammalian cell motility requires actin polymerization in the direction of movement to change membrane shape and extend cytoplasma into lamellipodia. Migration is believed to be central for primary tumor cells to reach blood vessels or lymphatics in order to metastasize [34]. In agreement with this, we find actin pathway is up-regulated in metastasizing tumors.

### 1.7 Signal transduction

S1P pathway is up-regulated according to GSEA. Sphingosine-1 phosphate (S1P) is a lysolipid, acting via cell surface coupled G-protein coupled receptors, and required for migration, proliferation and survival of breast cancer cells. Ectopic expression of the enzyme responsible for S1P production, SphK1, increases tumorigenity and angiogenesis and protect cell from apoptosis [35]. This supports our result, that increased expression of S1P pathway members is involved in metastasis.

Several other signal transduction pathways are regulated. The gene set GPCRDB_other, covers a group of G-protein coupled receptors targeted by a broad range of ligands, is down-regulated. Interleukin pathways (Il-3, Il-6, and Il-7) are also down-regulated possibly indicating decreased inflammatory response in metastasizing cells. Progesterone signaling pathway (MPRPATHWAY) is increased and ER transcriptional activity is enhanced according to motif analysis confirming that elevated sex-hormone load may lead to worse prognosis of breast cancer patients, which is supported by the widely use and benefit of anti-hormonal treatment of breast cancers retaining hormone receptors. On the other hand nuclear hormone receptors (NUCLEAR_RECEPTORS, table 2) theme self are down-regulated in metastasizing tumors in agreement with loss of ER and PgR as potent negative prognostic markers.

### 1.8 Proteasome pathway

Another pathway targeted for inhibitory treatment is proteasome, a strategy that is supported by our finding that the pathway is up-regulated in metastasizing tumors. Proteasomes normally perform controlled degradation of proteins and proteins selected for degradation by tagging with a poly ubiquitin chain. In addition, proteasomes are important regulators of several key regulatory proteins including p53, cyclins, CDK inhibitors and NF-kB. The proteasome inhibitor bortezomib has been suggested for treatment of several cancers including breast [36].

### 1.9 Immune system

Immune function is also impaired; tollpathway, forming part of innate immune system, is down-regulated in metastasizing cells (table 2). Toll-like receptors (TLR) are activated by pathogen expressed molecules and initiate immune response by release of pro-inflammatory cytokines. Activation of TLR's with synthetic agonists, have shown promising results for treatment of several cancers, by inducing apoptosis and elevating sensitivity of to cancer cells to chemotherapy [37,38]. The present results may support this strategy in breast cancer [37].

### 1.10 DNA damage sensing and repair

ATRBRCA pathway is up-regulated in poor outcome tumors. Several genes in this pathway are mutated in hereditary breast cancer (*BRCA1, BRCA2, ATM, p53*, Fanconi anemia genes, and *CHEK2*) and the pathway is believed to prevent cancer development by inducing cell cycle arrest, DNA repair and apoptosis after DNA-damage [39]. Up-regulation in metastatic cells may indicate a compensatory mechanism or malfunction of this pathway. Motif analysis indicate that BACH1, a transcription factor that interacts directly with BRCA1, is up-regulated [40]. Increased DNA repair has also been reported from Wang et al when inspecting their prognostic classifier genes [5].

### 2 Gene expression based pathway and transcription factor analysis

The present analysis of gene expression profiles of primary tumors identifies several pathways associated to clinical outcome. The identified pathway gene sets display significant imbalanced expression between metastasizing and non-metastasizing breast tumors across several data sets. This supports our hypotheses that several pathways are involved in breast cancer metastasis and that they are reflected in expression profiles of primary tumors. Furthermore, motif analysis demonstrates many significantly deregulated gene sets with common transcription factor binding sites. The transcription factor binding sites are identified by bioinformatics analysis and are for the most part not functionally validated [13]. The finding that these gene sets are significantly differentially expressed indicates that the individual genes in the gene sets are actually responding to the relevant transcription factors. Several of the motif gene sets associated to metastasis have been linked to certain pathways as discussed in the pathway sections. Some of the transcription factors have also previously been linked directly to survival. A prominent example is estrogen receptor targeted genes that are up-regulated in metastasizing tumors. Another well known transcription factor is Myc for which gene amplification is associated to poor prognosis [41]. Also transcription factor YY1 has been associated with metastatic potential in several cancers[19]. A number of predicted elements with unknown transcriptions factors are present in promoters of up-regulated genes. Further studies are needed to identify these factors and their functions. Interestingly, only up-regulated gene sets are identified in motif analysis, in agreement with our previous observation of a majority of up-regulated genes in metastasizing breast tumors [1]. Predominance of up-regulated pathways with both GSEA (26 out of 38 pathways) and GenMAPP (11 out of 19 pathways) meta-analysis supports this. The false discovery rate used for correction for multiple testing is dependent on the data structure and might bias this conclusion. However, the imbalanced distribution of significant pathway and motif gene sets is confirmed using Bonferroni method (data not shown). No miRNA targeted gene sets are identified in motif analysis which may reflect poor algorithms for prediction of binding sites.

Different outcomes are used in the included studies: relapse, metastasis, distant metastasis and death from breast cancer. This may bias the results because relapse (3 data sets) includes local recurrences and these may be result of suboptimal surgery or mechanism of spreading different from distant metastasis. Regional metastasis, i.e. recurrence in lymph nodes may also be the result of different metastasis mechanism compared to distant metastasis. However, local and regional recurrences constitute a minor fraction of a typical tumor-bank cohort, resulting in minor bias. To illustrate this, we examined a large Danish tumor-bank of primary tumors collected at Funen from 1989 to 1999. Among patients that experienced recurrences, 17% got local recurrence, 4% got regional metastasis, and 79% got distant metastasis (data from DBCG, ).

A varying fraction of the tumors in the individual data sets have disseminated cells to the lymph nodes. Classification of lymph node positive patients without recurrence as non-metastasis may be controversial. This may bias the results towards the metastatic mechanisms following primary spread to lymph node. Furthermore, a minor group of included patients have received adjuvant treatment that will bias the clinical outcome because a fraction of the patients are responding to the therapy. One data set, Huang, display conflicting results in pathway and motif analysis (table 2, 3, 4). Noteworthy, the patients in this study were all lymph node positive and received chemotherapy, which may explain the different results. Other subgroups of tumors e.g. molecular subtypes are also equalized by our approach resulting in identification of general mechanisms involved in metastasis.

Overall, the included studies mainly contain lower risk node negative untreated groups and the main outcome is distant metastasis. The noise from other groups and outcomes represented in the data sets may reduce the significance of identified pathways. This actually means that our statistical method is rather conservative. Furthermore, the statistical test is even more conservative because only ranking information from individual GSEA and GenMAPP analyses are included. Using actual statistical parameters from the analyses would increase the gathered significance. However, due to the different distribution of the parameters it was impossible to use them in meta-analysis.

Few other studies have investigated biological mechanisms in breast cancer gene expression data sets comparing metastatic and non-metastatic outcome. The approaches that have been used mainly concern the genes in outcome classifiers (typically below 100 genes) often including a large fraction of genes with unknown function. These studies have mainly reported cell cycle, growth and proliferation to be up-regulated, but some have reported angiogenesis, invasion and signal transduction [10], while others have identified cell death, DNA replication, recombination and repair genes [5] or motility [11]. However, the concordance in these findings besides cell cycle is low. We have in a previous study performed meta-analysis of genes present in classifiers and found cell cycle related gene ontology categories to be strongly overrepresented, while other categories were not present in more than one gene set [12]. Furthermore, classifier genes are very dependent of classification algorithm, definition of training and testing set, and many different classifiers may result in same classification performance [42]. Yu et al. [43] recently performed pathway analysis of 500 gene signatures generated by re-sampling training sets from the Rotterdam data set. In this way they minimized the bias from definition of training set, however, they collected the 100 most significant genes each time, ignoring biological mechanisms beyond the most strongly deregulated pathways.

Very different from this restricted approach this study utilizes GSEA including all genes in data sets, offering the possibility to reveal mechanism beyond cell cycle associated to metastasis. Furthermore, meta-analysis identifies gene sets with remarkably concordance between data sets. The findings are strengthened by concordant findings with GenMAPP utilizing only the most significant genes. Compared to the overrepresentation analysis often performed for classifiers, GenMAPP includes app. 500 significant genes improving the likelihood of identification of relevant biological functions. Disagreement between the two programs for some gene sets identified by one program but not the other may be explained by the different algorithms. GenMAPP may tend to ignore gene sets that are composed of genes with weak individual contribution, while GSEA can give these a high combined enrichment score if the fraction of these genes in a gene set is high. Another explanation for discordant finding is different definition of gene sets. Several GSEA gene sets are adopted from GenMAPP, but there are several other contributers [15].

Sorlie at al. has also used global gene expression patterns of large sample sets of breast tumors to investigate biological mechanisms in breast cancer [44]. However, they used a very different approach by performing unsupervised clustering of samples to identify new subgroups that subsequently were found to be associated with clinical outcome. Their results point at different progenitor cell types and estrogen and HER2 receptor status, but do not elucidate the different biochemical pathways involved in metastasis. Very different from this, we have not aimed at sub grouping tumors but instead intended to investigate general metastatic pathways. Several pathways are also supported by motif analysis indicating plausibly causal explanation for observed gene expression differences.

Many of the identified pathways and transcription factor have previously been identified with different focused techniques. By our global approach we have validated these findings. Furthermore, a number of new pathways and transcription factors and promoter elements not previous linked to metastasis are identified. Further validation and functional testing is required to confirm their role in metastasis.

## Conclusion

By performing pathway meta-analysis, we have identified several pathways involved in breast cancer metastasis. Cancer is a genetic disease, and somatic mutations and genomic instability are features of cancer development and progression. In agreement with this, we find DNA damage and repair pathway up-regulated in metastasizing breast tumors. Uncontrolled cell cycle, a feature of cancer cells compared to normal cells, also characterize metastatic cells compared to non-metastatic cells. Growth factors are often hypothesized to play central role in cancer proliferation and progression. We identify several changes in growth factor pathways: PDGF and EGF pathways are reduced, while signaling by VEGF, estrogen and progesterone, is enhanced. The high proliferation rate in tumors may lead to hypoxia, and in order for cancer cells to survive this, defense mechanisms are required and attraction of blood delivery is central. Changed metabolism towards glucolysis at expense of amino acids and lipids, help cancer cells to survive hypoxia, and angiogenesis improve blood delivery thereby reducing hypoxia. Angiogenesis also ensures a shorter distance for cells to the blood stream, and enhanced migration is essential to cover this distance. Immune system may protect against cancer progression supported by our finding that innate immune system is impaired in metastasizing tumors. Finally, proteasome pathway, an important regulator of proliferation and apoptosis, is impaired.

## Abbreviations

GSEA: Gene Set Enrichment Analysis; KEGG: Kyoto Encyclopedia of Genes and Genomes; MSigDb: The Molecular Signatures Database; FDR: False discovery rate

## Competing interests

The authors declare that they have no competing interests.

## Authors' contributions

MT and TK designed the study, QT developed methods for statistical analysis and MT performed data analysis.

## Pre-publication history

The pre-publication history for this paper can be accessed here:


